# Healthcare Professionals’ Perspective on Palliative Care in Intensive Care Settings: An Interpretive Descriptive Study

**DOI:** 10.1177/23333936221138077

**Published:** 2022-12-06

**Authors:** Hanan Hamdan Alshehri, Axel Wolf, Joakim Öhlén, Sepideh Olausson

**Affiliations:** 1 Institute of Health and Care Sciences, Sahlgrenska Academy, University of Gothenburg, Sweden; 2 University of Gothenburg and Region Västra Götaland, Sahlgrenska University Hospital/Östra, Sweden; 3 University of Gothenburg and Palliative Centre, Sahlgrenska University Hospital Region Västra Götaland, Sweden

**Keywords:** qualitative research, palliative care, end-of-life care, healthcare professionals, intensive care units, Saudi Arabia

## Abstract

There is a growing need to integrate palliative care into intensive care units and to develop appropriate knowledge translation strategies. However, multiple challenges persist in attempts to achieve this objective. In this study, we aimed to explore intensive care professionals’ perspectives on providing palliative and end-of-life care within an intensive care context. We used an interpretive description approach and interviewed 36 intensive care professionals at four hospitals in Saudi Arabia. Our findings reflect a discourse about end-of-life care driven by a do-not-resuscitate classification and challenges associated with family involvement in care goals. We provide key insights of importance for the development of strategies for the integration and knowledge translation of palliative care into intensive care contexts.

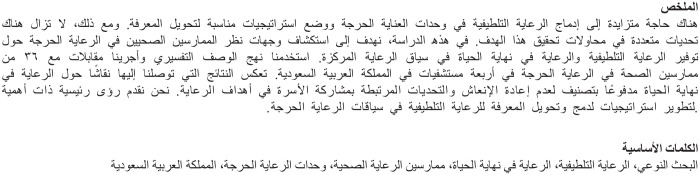

## Introduction

Intensive care units provide care for the most acutely and severely ill patients (Bardi et al., 2021; Elhadi et al., 2021). Patients may also be admitted to intensive care units because of serious or acute complications arising from chronic illnesses, such as trauma, intoxication, and sepsis (Hartjes, 2015). Therefore, end-of-life care is an unavoidable aspect of intensive care; for this reason, palliative care is increasingly recognized worldwide as an essential and integral component of intensive care (Aslakson et al., 2014). The latter is also proposed as a goal of care by the Intensive Care Society (Society of Critical Care Medicine, 2020).

### End-of-Life Care Versus Palliative Care

End-of-life care refers to care during a poorly defined period prior to death. This definition is descriptive and challenging due to the limitations of death prediction in the medical field (Meier et al., 2010). According to the WHO, palliative care is “an approach that improves the quality of life of patients and their families facing the problems associated with life-threatening illness, through the prevention and relief of suffering by means of early identification and impeccable assessment and treatment of pain and other problems, physical, psychosocial and spiritual” (World Health Organisation, 2020). Consequently, the objective of palliative care is to improve the quality of life and the well-being of the patient, their family, and their caregivers (Radbruch et al., 2020), which, in intensive care, may primarily focus on relieving the patient’s distress and supporting their families (Aslakson et al., 2014). Multidimensional care needs to be integrated to accomplish this objective (Wood et al., 2019). In addition, palliative care refers to care for patients with progressively life-limiting conditions, not only targeting the final dying phase but may also covering the months or years before death (Cruz-Oliver, 2017), although, in these cases, it is usually provided outside intensive care. Thus, palliative care is applicable to care at the end-of-life as well as earlier on in patients’ trajectories in cases with a high risk for mortality (Radbruch et al., 2020).

Two principal forms of palliative care are a consultative model and an integrative model, both of which have demonstrated their value in intensive care units (Nelson et al., 2013). In the consultative model, a palliative care specialist provides vital input in tailoring care and treatment for the patients in need of palliative care (Wysham et al., 2016). In the integrative model, intensive care clinicians are responsible for decision-making concerning palliative care and related issues such as symptom management. The latter relies on the assumption that all ICU clinicians have the necessary knowledge of and are responsible for patients in need of palliative care (Nelson et al., 2013).

Existing knowledge emphasizes that the aim of the integration of palliative care approaches in intensive care settings is to enhance dignity by stepping down life-saving invasive treatment strategies. In addition, it aims to prevent or treat pain, distress, and suffering for patients for whom a cure is no longer expected, regardless of age and diagnosis (Mercadante et al., 2018; Mularski et al., 2005). In this approach, the needs of family members are also considered, and the aim is to support them throughout this challenging period. Cultural disparities between patients, families, and professionals are incorporated and acknowledged in palliative care (Cain et al., 2018).

In this sense, palliative care can be regarded as a normative *quality mark* for care provision for patients and their families, while end-of-life care comprises the descriptive practices for care delivered to patients in intensive care units close to death (Rocker, 2010). Hence, while palliative care and end-of-life care are closely connected, palliative care is not limited to end-of-life care, especially in the case of chronic, deteriorating illness (Aslakson et al., 2014).

Saudi Arabian culture concerning death and dying is closely linked to the Muslim faith and emphasizes the provision of spiritual care for patients who are facing the end of their lives. A multi-method Saudi Arabian study by Tayeb et al. (2010) examined the perspectives of patients and health care providers and showed that spiritual support and a “good death” according to Muslim beliefs consist of three elements: (a) rituals related to faith, for example, having someone at the bedside to assist the patient with Shahada that is, the final testimony of the Muslim faith, reciting the Quran, and positioning the body toward Makkah; (b) self-esteem and appearance that is, the appearance of the body for the family and friends; (c) family assurance after the patient’s death (Tayeb et al., 2010). Another study investigating families’ experiences of being with dying patients reports similar findings, namely, that the spiritual support provided according to Muslim culture as part of end-of-life care is of crucial importance to patients’ loved ones (Al Mutair et al., 2020).

### “Do Not Resuscitate” Classification

Traditionally, intensive care has been a place of care for severely ill patients when the patient’s aims of curative therapy are recognized by the patient, family members, and/or intensive care professionals as no longer effective. *Withdrawal of life-sustaining treatment* is actively removing treatments that are required for the patient’s survival, such as ventilator support and vasoactive medicines (Hoel et al., 2014). Furthermore, “do not resuscitate” (DNR) is a common term used in intensive care clinical practice (Lo et al., 2020; Pastrana et al., 2008) indicating that a clinical instruction for cardiopulmonary rescue should not be initiated in the case of fatal failure in ventilation and/or circulation (Piryani & Piryani, 2018). A DNR classification is an important screening technique for identifying individuals who may not benefit from cardiopulmonary rescue and may have needs for palliative care, which may be assessed in collaboration with a palliative care specialist (Nelson et al., 2013). A DNR order can also be associated with the transition from curative care to palliative care (Fan et al., 2018; Piryani & Piryani, 2018), regardless of whether the patient is at end-of-life or not. Consequently, scholars have examined the link between palliative care and DNR in previously. For example, Taubert and co-authors have investigated the impact of education interventions for both healthcare professionals and patients in relation to the DNR order and its role in palliative care. It was found that DNR decision-making improved among healthcare professionals through the increased use of palliative care in curative treatments (Taubert et al., 2018). There is also evidence that DNR status may improve patients’ access to palliative care in the context of intensive care (DeCato et al., 2013; Wachterman et al., 2016).

### Challenges in Integrating Palliative Care in the Context of Intensive Care

These perspectives are important in designing knowledge translation strategies for increasing the integration of palliative care into intensive care. There is a need to explore practice perspectives on both palliative and end-of-life care to inform the development of knowledge translation strategies.

Palliative care is relevant to all critically ill patients admitted to intensive care units because it contributes to better outcomes, such as improved discharge processes, reduced length of stay, decreased rates of prolonged mechanical ventilation (Curtis et al., 2011), and lower medical cost (Huang et al., 2020). While the decision to discontinue curative and resuscitative treatments leads to death in intensive care units or transfer to a step-down unit or general ward for palliative treatment and care (Ma et al., 2019; Zalenski et al., 2017), ultimately, either will result in reducing the duration of stay in intensive care units (Bharadwaj et al., 2016; Mun et al., 2016) and in supporting intensive care clinicians in their decision-making (Lazenby et al., 2017).

Despite efforts to improve and integrate palliative care worldwide, it has not been successfully incorporated into intensive care units because many challenges persist in clinical practice (Nelson et al., 2010; Zaman et al., 2017). In a recent systematic review (Hamdan Alshehri et al., 2020), we found that organizational structures, work environment, family and patient involvement, and a lack of clear decision-making regarding prognostication and care transition are the factors that can influence the integration of palliative care into intensive care contexts. In this review, we identified few studies that have investigated palliative care approaches in intensive care units from the perspective of healthcare professionals. In the present research, we seek to address this gap by exploring intensive care professionals’ perspectives on the provision of palliative and end-of-life care within intensive care units.

## Methods

We selected an interpretive description design due to the practice-oriented aim of our study. Interpretive descriptive design is tailored to generating knowledge about complex clinical practice phenomena (i.e., in this study, palliative and end-of-life care in the context of intensive care) through naturalistic and constructivist inquiry (Thorne, 2016). Given the context of the study and complexity of these concepts, we believe that an interpretive description design provides access to intensive care professionals’ perspectives on the provision of palliative care. With this design, we can inquire and thus identify experiential meaning patterns from the participants’ perspectives while simultaneously verifying variations between them (Thorne, 2016). As Thorne (2016) argued, “nursing epistemology is always concerned with both individuals’ human experiences with knowledge that can be derived from the population” (p. 6). Therefore, in developing knowledge that is applicable in nursing and interprofessional intensive care practice, we need to generate an improved appreciation of relevant practical problems of palliative and end-of-life care in intensive care contexts (Thorne, 2016; Thorne et al., 2016).

### Setting and Sample

We conducted our study in the Kingdom of Saudi Arabia, whose Ministry of Health healthcare system is comprised of two main healthcare providers: the government sector and the private sector. Currently, the system includes three levels of care: primary, secondary, and tertiary. These care levels are provided by healthcare centers, general hospitals, and specialist hospitals. The total number of beds available in all Ministry of Health hospitals is 13.1 per 1,000 inhabitants (Ministry of Health, 2018). The total number of beds for adult intensive care is 2.5 per 1,000 inhabitants, of which 628 beds are found within the metropolitan area of Riyadh, which has approximately 7.7 million inhabitants (Al-Omari et al., 2015; Ministry of Health, 2018); this is the area where we collected the data (see Table 1). Nurses’ education in the Kingdom of Saudi Arabia includes a 4-year bachelor’s degree in nursing, followed by a 1-year internship and various post-graduate healthcare programs, including the adult critical care sub-specialty (provided by the Saudi Commission for Health Accreditation).

**Table 1.** An Overview of Types of Critical Care Level Included in the Study.

Critical care level	Primary level hospital	Secondary level hospital	Tertiary level hospital
Total bed capacity	>50 beds	>100 beds	Medical cities
Level description of critical care	*Limited equipment *Limited ICU education and training *Located in villages and remote areas	*Equipped with ICUs and larger equipment *Intensive care specialists available *Located in small cities	*Specialized ICU with advanced equipment *Certified intensive care specialist available 24 hours/7days *Located in big cities

We conducted our study in four governmental hospitals (designated Hospitals A, B, C, and D) in metropolitan Riyadh, Saudi Arabia. The Ministry of Health oversees these hospitals, which provide two of the three levels of care (Marshall et al., 2017). Hospitals A and B operate in tertiary intensive care units (containing 48 and 100 intensive care beds, respectively), whereas Hospitals C and D operate in secondary intensive care units (containing 60 and 16 intensive care beds, respectively) (see Table 1). These hospitals also offer various specializations (see Table 2).

**Table 2.** Overview of the Hospital Setting.

Hospitals	Service level	Number of healthcare specialties in each hospital
Specialist hospital A	Tertiary	*Physician 5*
		*Nurses 8*
		*Total 13*
Specialist hospital B	Tertiary	*Physician 2*
		*Nurses 5*
		*Respiratory therapists 3*
		*Total 10*
General hospital C	Secondary	*Physician 1*
		*Nurses 3*
		*Respiratory therapists 1*
		*Dietician 1*
		*Total 6*
General hospital D	Secondary	*Physician 4*
		*Nurses 3*
		*Total 7*

We applied a purposeful sampling approach in which we included participants from different professional backgrounds to generate data about variations and nuances of the studied phenomenon. The inclusion criteria consisted of working as a bedside healthcare professional and being able to speak and understand the English language. We invited professionals with Saudi and non-Saudi nationalities within different intensive care specialties to enhance the credibility of our study (Thorne, 2016; Thorne et al., 2016).

A total of 36 professionals participated in the study: 19 nurses, 12 physicians, 4 respiratory therapists, and 1 dietician (see Table 3). All participants worked directly with patients in a bedside role in adult intensive care. Currently, there is a shortage of qualified intensive care professionals in Saudi Arabia; therefore, intensive care personnel typically include a large number of non-Saudi and several Saudi healthcare professionals. The sample reflected this.

**Table 3.** Characteristics of the Study Participants.

Characteristics	Number of participants
Healthcare professions	
Nurses	(19)
Physician	(12)
Allied health professionals*	(5)
Gender	
Male	(15)
Female	(21)
Nationality	
Non-Saudi	(23)
Saudi	(13)
Highest degree held	
Bachelors	(25)
Master	(8)
PhD Degree MD	(3)
Specialty education	
Critical care specialties	(14)
Pulmonary	(1)
Cardio critical care	(1)
Anesthesiology	(1)
Neurosurgery	(1)
Trauma care specialty	(1)
None	(17)
Type of intensive care	
General intensive care	(27)
Medical intensive care	(4)
Neuro intensive care	(4)
Surgical intensive care	(1)

* Allied health professionals including dietitian and respiratory therapies.

### Data Generation

The first author contacted the head of each intensive care department and provided information on the study. The unit managers and head nurses subsequently introduced the first author to the intensive care staff, and potential participants were those who were present at the units on these days. The first author met bedside professionals in the intensive care unit and presented key background information, including the purpose of the study, to each of them individually prior to their participation. There was no relationship between any of us (the authors) and the study participants. With respect to the nature of the work in intensive care units, we scheduled the interviews and conducted them in accordance with the participants’ preferences. However, we interviewed most participants during their breaks. This was the case for nurses and allied healthcare personnel, including respiratory therapists and dieticians. In addition, a medical secretary in each hospital sent emails to the physicians and verified the interview schedules and locations for all physicians and consultants who agreed to participate. The first author developed a plan for performing interviews in the fourth hospital based on bed capacity and personnel. For example, the first author recorded information regarding Hospitals A and B and the schedules of the interviews in the planning notes because the geographical locations differed between the hospitals.

The in-depth, face-to-face interviews comprised open-ended questions focusing on the practice of palliative and end-of-life care within intensive care units. We piloted the interview questions prior to the principal interview stage with three staff nurses working in intensive care units (outside the hospitals chosen for the study). We did not make subsequent revisions to the interview guide, and we did not include the pilot interviews data in this study. The interview guide included the following three questions: (1) *Can you please tell me about how you provide care for intensive care patients who need palliative care or are about to die?* (2) *In your experience, what was it like to provide care for the family of a patient who is in need of palliative care or is about to die in intensive care units?* (3) *How did you feel when you cared for a patient who was about to die and for their family?* We asked follow-up questions (*Can you please tell me more? Could you provide an example?*) to clarify the participants’ initial answers, thus enabling us to capture a broader perspective of their experiences within the scope and aim of the study (Brinkmann & Kvale, 2015). We conducted the interviews in the English language in an intensive care conference room during the participants’ regular working daytime hours. We audio-recorded and transcribed the interviews verbatim, with additional field notes taken to record the interview details and locations. The interviews ranged in length from 12 to 59 minutes (median = 39 minutes). In addition to the interviews, we used a field notebook and a reflective journal to ponder the answers provided by each group of intensive care professionals.

### Data Analysis

We analyzed the data according to the interpretive description approach and applied constant comparative analysis, as suggested by Thorne (2016). At the preliminary stages of analysis, our emphasis was on obtaining a broader view of the data in relation to the research phenomenon, which is the provision of palliative and end-of-life care for patients in intensive care as experienced by professionals. The first author read each interview several times alongside relevant written notes. Having become familiar with the data, the first author then focused on the research questions and developed a more concrete data-processing strategy consisting of the following steps. The authors used the NVivo software program in order to work efficiently with the data. The program supported the organizing, coding, sorting, and retrieving data segments across interviews and thus facilitated analysis of similarities and differences, including visualizations of, for example, a code tree for each of the participating professions (nurses, physicians, allied health care professionals), comparing them with each other and also linking the quotes that supported coding of the data (Dollah et al., 2017). It also assisted us in tracing back and double-checking the data through various stages of analysis. Furthermore, throughout the analysis, analytic questions were asked in order to facilitate interpretations grounded in the data.

First, the first author broadly coded the data using NVivo, version 11, leading to the generation of larger text segments relating to how palliative and end-of-life care were provided. These also reflected the perceived consequences of integrating palliative care into intensive care for patients and their families. Second, the first and last authors highlighted the differences and commonalities among participants regarding their perspectives on providing palliative and end-of-life care in intensive care units. Third, the first and last authors mapped initial codes, grouped them, then subjected the data to a continuous comparative analysis process. Finally, the first and last authors worked with each broad code grouped under a theme that reflected the meaning of the broad codes. Altogether, we meticulously reviewed and discussed the findings on numerous occasions until all aspects of the analysis were integrated and elaborated on.

### Ethical Considerations

We obtained ethical approval from both the Ministry of Health Research Centre and the ethics committees of the four hospitals. We developed a study protocol in accordance with the ethical principles outlined by the Helsinki Declaration (World Medical Association, 2020). We observed ethical rigor by ensuring that the participants received oral and written information about the study and that they understood that their participation was entirely voluntary and subject to the provision of written consent. They knew that they had the right to withdraw at any stage without needing to justify their decision. The hospitals’ ethics committees (Institutional Review Board No. H1RE-14-may-19-01, 18-499E) and the Ministry of Health Research Centre (ID numbers: 1440-238565, 1440-225577, 1440-225751, and 1440-226192) approved our study.

### Rigor

In this study, we followed Thorne’s (2016) recommendations for reporting qualitative research. Before starting the data collection, we critically reflected on our prior understanding of the process to adopt a more critical attitude throughout. The first author was a doctorate candidate, and all co-authors are experienced researchers with strong knowledge of research methods and are employed within the fields of palliative and critical care. Furthermore, we considered credibility during the study process. We reinforced this by double-checking the data collection process, including our perspectives as researchers. We asked each participant to elucidate their answers when we needed greater clarity during the interviews. We took notes during each interview to ensure that each participant addressed all the questions and that every interview was preceded by a briefing on participant diversity, including their backgrounds.

Representative credibility also provided logical verification of the data in terms of their accuracy, relevance, and significance. This was ensured by the similarity between two or more independent participants, but differences were also considered part of the application of the interpretive description method in this study (Thorne, 2016). This means that the findings emanating from a qualitative inquiry might be consistent if the research is undertaken repeatedly and draws on the same participants, codes, and context. In addition, prolonged engagement with the data, keeping study notes and diaries, having regular supervision meetings, and confirming data coding were strategies used to support the credibility of the findings.

In this study, establishing a relationship between the data, analytic methods, and results so that readers may recognize the adequacy of the findings is regarded as analytic logic. To this end, we evaluated the consistency of the study processes and critically discussed this in team meetings. We conducted the study in four different hospitals and intensive care units and applied purposeful sampling to consider the diversity of intensive care professionals’ experiences and perspectives, as well as the study context.

## Findings

We interviewed a total of 36 participants comprising registered nurses (*n* = 19), physicians (*n* = 12), and allied health care professionals (*n* = 1 dietitian and *n* = 4 respiratory therapists) regarding their experiences and perspectives related to providing palliative and end-of-life care to patients within intensive care units. Overall, we found that while palliative care has yet to become part of a systematic foundational approach, there are standardized structures in place in relation to DNR policies and end-of-life care in the various intensive care units in which the participants were located. We identified support actions and associated challenges for the provision of palliative care within the context of intensive care. We present and structure the findings according to three broad themes reflecting the various perspectives held by intensive care professionals: (1) a tendency to equate palliative care with end-of-life care driven by a DNR classification, (2) decision-making regarding the goals of care centering on DNR classification, and (3) challenges associated with family involvement in care goals.

### A Tendency to Equate Palliative Care with End-Of-Life Care Driven by a DNR Classification

The participants’ perceptions of palliative care were largely similar to their perceptions of end-of-life care and were predominately related to and exemplified by classifying patients according to their DNR status and to current policies. In this way, DNR was the primary input for most of the participants in the interviews and was essentially equated to both end-of-life care and palliative care. Moreover, patients’ diagnoses seemed to be central to relating to palliative care provision. When intensive care professionals reflected on their experiences of caring for patients at end of life, they frequently referred to cases in which patients had cancer diagnoses, as exemplified the participant comment: “Primarily, stage 4 carcinoma patients with organ failure are allocated a DNR status. In addition, very old patients, patients with poor GCS scores, and coma-stage patients are provided with palliative care only” (N-14).

Subsequently, the type of care provided to patients toward the end of their lives in intensive care units was determined by their diagnosis and type of DNR classification. We identified two major types of DNRs in the data. Specifically, most of the participants distinguished between *DNR with escalation* and *DNR without escalation*. DNR with escalation meant the provision of all intensive care treatment and support, except cardiopulmonary resuscitation (CPR). DNR without escalation was described by most participants as the complete absence of treatment:

*We have DNR with escalation, which means we can still give medications if the patient will deteriorate and if the blood pressure will go down; if the patient needs it, we can also intubate and hook them to the ventilator to support their life, to prolong it. DNR without escalation means if a patient deteriorates, we will not give anything except normal saline and IV fluids. (N-7)*

Furthermore, some participants precisely explained DNR, expounding on whether the patient should be offered a *DNR code*: “The basic DNR is simply not to do CPR, do not defibrillate” (MD-1).

When we invited the participants to talk about their experiences of providing palliative care and end-of-life care in intensive care contexts, both nurses and physicians emphasized that pain management and bodily care were essential elements and that these were related to a DNR decision. One nurse stated, “If this patient is an end-of-life case, we mostly only control the pain. We give some medications that provide comfort for the patient. This is because they are DNR” (N-1). Only a few participants emphasized aspects relating to the spiritual and psychological dimensions of care. Nurses also described the basic nursing care provided as being fundamentally the same regardless of whether the patients had reached an acute disease stage or were at end-of-life. The physician participants expressed that intensive care patients often did not receive the appropriate palliative care and that the focus was oncology patients; the best option was stated as referring these patients to their respective hospitals’ palliative care departments. According to the physicians, however, this rarely happened and was limited to patients with cancer.

### Personal Views of Hospital Policies

From the participants’ narratives, we can identify a clear gap between providing palliative care focused on end-of-life care and palliative care focused on DNR policies. From the data, we observed that despite policies and DNR guidelines being in place, there were significant differences in practice and care provision described among intensive care professionals, depending on their personal beliefs and feelings. In the physician data, emphasis was given to the participants’ awareness of the types of DNR orders and reference to hospital policies in relation to these orders. However, they provided care directed toward achieving the goal of intensive care provision (i.e., providing life-supporting interventions). The participants highlighted that intensive care professionals may unintentionally provide life-saving interventions, despite hospital policies. We identified the motive from the following line: “we must give everybody a chance” (MD-2).

Some physicians approached care for patients whose deaths were imminent in a similar way. They explained that they provided maximum care, at least initially, regardless of the patient’s condition, even if a DNR order was justified.

*Generally, taking care of patients who are about to die is more difficult. There is a need for practitioners to be careful and to check all the details. We should act quickly to try to prevent the patient from dying, if possible. (MD-8)*

Many of the nurses shared the same view as physicians regarding maximum life-supporting interventions. We can interpret this as a moral and ethical standpoint: “We don’t want any patient to die in our care. I think this is the same concern faced by all nurses” (N-15).

Although some of the nurses believed that intensive care nursing should incorporate a clear policy regarding care plans for end-of-life patients, they felt disappointed with most of the DNR decisions and regarded them as breaches of care trajectories. For example, *one nurse said, “We are not providing CPR and cardiogenic shock like that, but we are still giving the inotropes like that. The situation with DNRs is very confusing” (N-10).*

### Decision-Making Regarding the Goals of Care Centering on DNR Classification

The participants expressed that end-of-life care decision-making processes involved assessing patients’ eligibility for DNR and assuming responsibility for care objectives. Some physicians stated that a combination of subjective and objective assessments was usually done when identifying patients who were eligible for a DNR code in intensive care units. While assessment tools, such as the Sequential Organ Failure Assessment and Acute Physiology and Chronic Health Evaluation IV (Apache) scores, were reported to be used to determine whether patients were eligible for DNRs or were at the end-of-life stage, only a few physicians discussed these tools. For example, one physician reported the following:

*It has an objective part and a subjective part. The objective part usually includes the severity criteria at this hospital. For example, we use the Apache IV score. Apache IV is a severity metric. There is also a mortality component and a clinical manifestation element. (MD-4)*

The assessments were described differently in intensive care settings. For example, most nurses and physicians working within neurological intensive care units described the computed tomography (CT) brain scan as an important assessment tool in the evaluation of patient eligibility for a DNR order. Furthermore, the participants identified patients as end-of-life when they presented with multiple organ failure, chronic illness, aging, and poor prognoses. “Here, chronic patients with poor prognoses are considered for DNRs – for example, end-stage renal disease with . . . ischemic stroke or poor CT scan results, along with the ESRD . . . they are considered suitable candidates for a DNR . . . sometimes” (N-9).

The nurse participants sometimes claimed that nursing assessments could offer guidance in identifying patients eligible for DNR in intensive care contexts. Some nurses stated that they used the Glasgow Coma Scale (GCS) as an assessment tool to identify these patients: “We check the Glasgow Coma scales GCS to see the score” (N-3). Nevertheless, the nurses stated that the identification of patients eligible for DNR was also done by physicians in intensive care: “DNR cases are identified by our consultant, as well as the primary physician looking after the patient, plus another physician” (N-15).

Despite views equating end-of-life care to palliative care and operationalizing it to a DNR status, views regarding the identification of patients requiring DNR varied. For example, nurses and allied healthcare professionals generally consider it to be a medical decision that should be solely the responsibility of the physician. This was highlighted in comments such as “It is the doctor’s decision.” Thus, the participants emphasized the importance of including three consultants in the assessment and confirmation of the DNR decision:

*In general, decisions relating to a patient who is about to die or whether they should have a DNR order [are] performed not by one physician alone but by three consultants. Of these three consultants, one will be the primary physician, one will be from the intensive care unit, and the remaining consultant will be from another specialty. The three must discuss the case together. (MD-8)*

Providing care for patients with DNRs presents challenges for intensive care professionals. Some nursing staff reported instances in which physicians altered directives or cancelled DNRs: “Physicians make a decision to issue a patient with a DNR. Then, later, after a few weeks, they cancel the DNR” (R-4).

## Challenges Associated With Family Involvement in Care Goals

The challenges identified were both related to communication and to family members’ emotional burden.

### Communicating Care Decisions With the Family

The identified challenges in supporting families included communicating the patient’s care goals and the difficulties that the family faces while caring for the patient at end-of-life. All the participants explained that their most challenging experiences involved dealing with the families and informing them about the patients’ conditions. Some challenges highlighted and discussed by the participants were family acceptance and ways of communicating with and supporting the family, which included the emotional responses evoked when the patient was at end of life (which was practically explained as having been assigned a DNR code). A recurrent theme in the narratives was families seeking the continuation of intensive care and life-supporting interventions and their lack of knowledge regarding the goals for medical treatments. We observed conflicting views in our data regarding communication and family involvement in the care objectives. For example, decisions regarding DNR codes could be influenced by the wishes of the family: “If the family does not agree to the DNR, we cannot implement it. We will provide every treatment for the patient” (MD-7).

However, a few physicians also believed that involving families in decision making was improper because the decision should be purely medical:

*No, it’s not ethical to give them that [decision] . . . to put that burden on [them] . . . Usually, the burden is on the physician and the medical team, and we just inform [the family]. The signature shows that they were informed about the situation and what the medical team will do . . . It’s not an agreement for a DNR; it just shows that they were informed about the DNR status. We don’t give . . . the family the burden of agreeing. (MD-4)*

### Facing Families’ Emotional Burden

Many of the participants described being challenged by the family’s emotions in numerous ways, and they used words such as *sad*, *angry*, *violent*, *very anxious*, *out of control*, *tense*, and *aggressive* to describe their experience. On many occasions, they narrated that families can become emotional when they learn that their family member requires a DNR order. One physician said, “We have difficulties with families sometimes because they will be angry if we just decide on the DNR for the patient” (MD-9). A nurse also stated, “Some people are very out of control in their emotions. That is the main challenge. We cannot face them; that is the main challenge . . . Just to provide good support” (N-12). Many nurses stressed that engaging with the family and their concerns about the patient’s condition during everyday care was the most difficult part of their roles as bedside nurses. This was due to ambiguity regarding who was responsible for providing information, insufficient communication with the family, and, sometimes, not being entirely honest about the care goals and updates on the patient’s condition. According to the nurses, decisions regarding care were not always communicated to family members, and they believed that the physicians were responsible for communicating the decision with the family first. One nurse said:

*It’s difficult to work with family members since they are shocked that we are doing nothing for DNR patients and that they are uninformed about the care decision or not. I’m not sure if they’ll be invited to the family meeting or if the doctors will meet with them. (N-10)*

## Discussion

In this study, we explored the perspectives of intensive care professionals on providing palliative care and end-of-life care in intensive care units, and this was reflected in the thematic structure of the findings presented. The perceptions held regarding palliative care and end-of-life care were clearly related to a DNR discourse and not to a palliative care discourse characterized by strategies to increase patients’ wellbeing and quality of life and relieving symptoms, distress, and suffering. The challenges associated with family involvement in end-of-life care mainly related to communicating care decisions with the family and facing the family’s emotional reactions.

### End-of-Life Care Framed by Critical Care Culture

The culture of critical and intensive care also appears to frame end-of-life care because we found no clear differentiation between palliative care and end-of-life care in our data. Thus, we found it difficult to conceptualize a palliative care culture in our findings. Nevertheless, we learned about the challenges of non-escalation or phasing out medical interventions, such as CPR, inotropes, and venting (in accordance mainly with the DNR policy rather than end-of-life care). We established that the care practices reported refer to the DNR policy rather than to the provision of end-of-life care and perhaps even less to palliative care in the context of intensive care. This contradicts the idea of providing palliative care for the increased well-being of patients and their families, which does not require any focus on DNR orders in end-of-life care within an intensive care setting (Aslakson et al., 2014). Furthermore, as there is no distinction between the descriptive term *end-of-life care* and the normative notion of palliative care in the results, designing knowledge translation strategies for palliative care integration into intensive care context is challenging. The perceived overlap of end-of-life care and a DNR status without relating to the enabling of well-being inherent in palliative care in our results will constitute a significant foundation for informing knowledge translation strategies for palliative care to become systematically integrated into intensive care units.

In this study, we found that the factors influencing the experiences of intensive care professionals were influenced by the culture espoused in their work setting, which primarily aims to save patients’ lives. For example, regardless of the patient’s state or whether the patient was assigned end-of-life care, the participants in this study reported an emphasis on delivering maximum life-supporting interventions to all intensive care patients. This finding contrasts with the perspectives of intensive care nurses in the United States, who have been found to be of the opinion that treatments and procedures take considerably longer than necessary and that nurses are *harming* patients by continuing to treat them rather than allowing them to die of their conditions (Stokes & Zoucha, 2021). In addition, the participants in this study rarely highlighted the spiritual and psychological care aspects; they instead placed greater focus on physiological needs, such as treating pain and other symptoms pharmacologically. This finding is unexpected because the culture in Saudi Arabia is known to target or emphasize the spiritual dimensions of palliative care (Al-Shahri, 2016; Tayeb et al., 2010). The same finding is consistent with that in a survey study of Czech nurses investigating end-of-life care activities, showing that nurses focused on bodily comfort needs when providing end-of-life care (Kisvetrová et al., 2017). A recent qualitative interview study conducted in Finland, (Haavisto et al., 2021) similarly found that nurses did not emphasize spiritual aspects when caring for patients.

### Communicating and Practicing Goals of Care

Assessments and decision-making responsibilities are important for the prioritization of a patient’s goals of care, and in our study, they are primarily associated with end-of-life care related to the DNR status and for patients with cancer as an underlying condition; the participants in our study expressed that palliative care remains primarily associated with cancer patients. This perception of care provision in intensive care units subdivides patients into those with DNR eligibility and those with cancer, which differs from the internationally suggested use of palliative care (Radbruch et al., 2020). The conclusions emerging from our study indicate that oncology is more intimately connected with palliative care at the end-of-life stage in an intensive care setting, at least in the Saudi Arabian health care context. This finding concurs with views on palliative care being equal to end-of-life care held by solid tumor oncologists in the United States (LeBlanc et al., 2015). This indicates the need to develop a common agreement on the concept of palliative care that can improve the care practices designed to support all patients requiring palliative care in intensive care contexts, regardless of the diagnosis and the decision-maker.

The experiential descriptions related to end-of-life care were driven by a DNR classification; however, the intensive care professionals in our study perceived DNR policies in end-of-life care in intensive care units as challenging and ambiguous, partly related to family involvement and partly to the DNR as a purely medical decision dependent on the consultant in charge of each case, thus yielding a variety of treatment outcomes. This lack of clarity was exacerbated when physicians modified or cancelled existing DNR orders. One reason for this could be the strong cultural norm disclosed by the participants in this study to prevent the death of a family member, indicating the presence of conflicting views between family wishes and care directives and policies. We can reasonably assume that there are similar cultural norms relating to preventing death that justify the use of a DNR code at end of life while simultaneously providing life-sustaining interventions because of the challenges of supporting families when one of their family members is at end-of-life in an intensive care unit.

### Challenges Involved in Communicating With Patients’ Families

The participants described how family members expressed emotions when informed about the patient’s care goal, particularly when they were told that the patient would be receiving end-of-life care. This is in line with the findings from previous studies examining the perceptions of the families of adult patients in intensive care units regarding decision-making about the goals of care (Kisorio & Langley, 2016). Families tend to struggle with emotional distress associated with the goals of care transitioning from receiving lifesaving efforts to focusing on end-of-life care (Hickman et al., 2016; Kisorio & Langley, 2016). Notably, when adopting a palliative approach, emphasis is supposed to be placed on how to increase wellbeing and relieve symptoms, distress, and suffering. Such examples were not given by the participants in this study. Nonetheless, in our results, emphasis is placed on including families in patient care by communicating with them and involving them in decision-making regarding the goals of care. This could be interpreted as promoting support for family members in intensive care units (Mitchell et al., 2016). However, some nurses mentioned the lack of information about a patients’ conditions available to families and the limited communication about the goals of care as sources of increased work-related stress for them, especially when families sought updates from them regarding a patient’s condition and care. Generally, involving families was considered challenging, and intensive care professionals expressed hesitation in including them because they did not want to burden the families excessively. Our results showed that, while a stronger emphasis on bedside care plays a significant role in the integration of palliative care into intensive care, much effort is being invested to support the families of patients. This support includes acceptance coaching about the goals of care, provision of information, facilitating family acceptance, and providing communication assistance. However, some of the nurses and physicians participating in this study also stated that the families of patients in the end-of-life period insist on life-supporting intensive care treatment instead of palliative care. This contrasts with the results of two previous studies conducted in non-Arab countries indicating that families in Western countries prefer palliative care over aggressive treatments for end-of-life patients, since they perceive the latter as indicating lower care quality (Ersek et al., 2017; Wright et al., 2016).

### Strengths and Limitations

We acknowledge several limitations of our study. First, the sample consisted of fewer native Saudi Arabian participants compared with non-native Saudi Arabian participants. We consider this a limitation because non-native Saudi Arabian participants may have less understanding of the Saudi Arabian healthcare context (e.g., organization, policy, culture) and may even lack knowledge or have a different understanding of the phenomenon under investigation. We used the English language in this interview study because it was spoken by the majority of healthcare professionals in Saudi Arabia (including the intensive care units); nevertheless, this may limit the scope of the study because participating in an interview in one’s first language can facilitate ease in the sharing of experiences and the level of detail in the accounts. Second, we conducted the study in one directorate in Saudi Arabia, and there may be value in seeking the views of other intensive care providers, such as private hospitals, to obtain a broader geographical representation and variation. Nonetheless, we made every effort to achieve sample diversity by involving several types of intensive care contexts and including various professionals in an intensive care team. Furthermore, we had an opportunity to scrutinize the data in a manner that aided the generation of variation and nuance in the findings in line with adopting an interpretive description approach. Using NVivo assisted us in managing large amounts of data in a time-saving and efficient way.

## Conclusion

In this study, we found that intensive care professionals encounter multiple challenges in interpreting end-of-life care policies and in understanding and applying palliative care within intensive care context, especially in using palliative care as a proactive approach and not only applying it in end-of-life care. DNR decisions and related discourse appear to be both a gateway to identifying and meeting needs for end-of-life care but also an obstacle to the recognition of needs for palliative care. Integrating palliative care systematically into practice and into the training and education of intensive care professionals is therefore essential to ensure the quality of care. The findings of our study can enrich and inform the development of appropriate and effective knowledge translation strategies when integrating palliative care into intensive care units. Additional studies are needed to explore how the challenges associated with patients who have actual or potential palliative care needs and their families can be successfully managed.
